# Differentiation of Human GBM From Non-GBM Brain Tissue With Polarization Imaging Technique

**DOI:** 10.3389/fonc.2022.863682

**Published:** 2022-04-28

**Authors:** Yi-Rong Liu, Hong-Hui He, Jian Wu

**Affiliations:** ^1^ School of Medicine, Tsinghua University, Beijing, China; ^2^ Tsinghua Shenzhen International Graduate School, Tsinghua University, Shenzhen, China

**Keywords:** polarization imaging technique, 3×3 Mueller matrix, clinical human GBM tissue, polarization parameters, frequency distribution histogram

## Abstract

As for optical techniques, it is difficult for the 5-aminolevulinic (5-ALA) fluorescence guidance technique to completely detect glioma due to residual cells in the blind area and the dead angle of vision under microscopy. The purpose of this research is to characterize different microstructural information and optical properties of formalin-soaked unstained glioblastoma (GBM) and non-GBM tissue with the polarization imaging technique (PIT), and provide a novel method to detect GBM during surgery. In this paper, a 3×3 Mueller matrix polarization experimental system in backscattering mode was built to detect the GBM and non-GBM tissue bulk. The Mueller matrix decomposition and transformation parameters of GBM and non-GBM tissue were calculated and analyzed, and showed that parameters (1−***Δ***) and ***t*** are good indicators for distinguishing GBM from non-GBM tissues. Furthermore, the central moment coefficients (CMCs) of the frequency distribution histogram (FDH) were also calculated and used to distinguish the cancerous tissues. The results of the experiments confirmed the feasibility of PIT applied in the clinic to detect glioma, laying the foundation for the subsequent non-invasive, non-staining glioma detection.

## 1 Introduction

Glioma is a type of malignant primary brain tumor that originated from glial cells. Tumor invasiveness makes it difficult to distinguish tumors from normal brain tissues visually, and the residual invasive cancer cells always lead to glioma recurrence and a negative impact on overall survival. Therefore, it is critical to distinguish between the glioma and normal brain tissues in glioma surgery; preoperative and intraoperative imaging methods can help determine the location of glioma and improve the resection rate of glioma while preserving the brain function of the patients (1). Clinically, the intraoperative imaging methods of glioma include ultrasound (US), magnetic resonance imaging (MRI), and various other optical techniques. However, MRI has low specificity and cannot completely identify the residual invasive glioma cells (2–4), and the use of intraoperative MRI may prolong the operative time and increase the risk of infection (3). The identification of glioma by US, although being cost-effective and easy to operate, depends mostly on the experience and skill of surgeons. As for optical techniques, it is difficult for the 5-aminolevulinic acid (5-ALA) fluorescence guidance technique to completely resect glioma due to residual cells in the blind area and the dead angle of vision under microscopy.

In fact, the occurrence and development of glioma can be characterized by different optical features from the normal brain white matter; therefore, in this paper we applied a new technique to detect glioma: polarization imaging technique (PIT). PIT is a potentially powerful and non-invasive method that carries rich microstructural optical information about biological tissues (5–8) and can be used to detect pathological changes (6, 9). The detected sample’s polarization information is carried out in a Mueller matrix (10–12), which has shown to be a comprehensive description and potential diagnostic tool for various complex tissues, and has been widely applied on biomedical diagnosis, especially cancer detection (13–21). Furthermore, conventional polarimetry combined with interferometry (22) techniques is promising to assess the 3D morphology of biological tissues and help confirm diagnosis of the disease (23).

PIT can provide optical difference and useful contrasts between the lesion and the healthy region when it is insufficient to be observed using conventional unpolarized-light-source intensity imaging methods. In the past few years, based on histological examination of the detected sample as the gold standard, various PIT images of cancers have been reported to differentiate between healthy and cancerous tissue (13, 24–30). In particular, tissue polarimetry was used to effectively analyze how the myocardial tissues were affected by disease for definitive diagnostics in forensic medicine (31). Machine learning algorithms were used to extract particular features for human *ex vivo* colon specimen classification between healthy and tumor zones (32). As for the nervous system, PIT combined with optical correlation tomography (OCT) has been reported to distinguish brain white matter and glioma tissues (33), which confirmed the feasibility of PIT being clinically applied to detect glioma.

In this paper, it is proposed that different polarization features are obtained to distinguish between GBM and non-GBM brain tissue using PIT combined with frequency distribution histogram (FDH) distribution and high-order statistical moments analysis. Specifically, we introduced the experimental setup based on backward scattered 3×3 Mueller matrix measurement without the circular polarizations; it significantly simplifies the experimental geometry, which is particularly appropriate for clinical PIT measurement (34, 35). It also allows the measurement of the clinical GBM tissue bulks. Then, applying it on the detection of GBM and non-GBM brain tissue, the PIT parameters (1**−*Δ***) and ***t*** were calculated using the Mueller matrix polar decomposition (MMPD) and Mueller matrix transformation (MMT) techniques (36, 37), which confirmed that parameters (1**−*Δ***) and ***t*** can be good indicators for distinguishing GBM from non-GBM tissues. Furthermore, we conducted FDH distributions and central moment coefficients (CMCs) (38) to examine in detail the dominant differences of normal and glioma tissues from a statistical perspective.

The determination of the different polarization characteristics of GBM and non-GBM brain tissue lays the foundation for the subsequent intraoperative PIT detection, and it is expected to make up for the shortcomings in clinical applications of the other imaging methods mentioned above.

## 2 Materials and Methods

### 2.1 Polarization Imaging Experimental Setup

**Figure 1:**
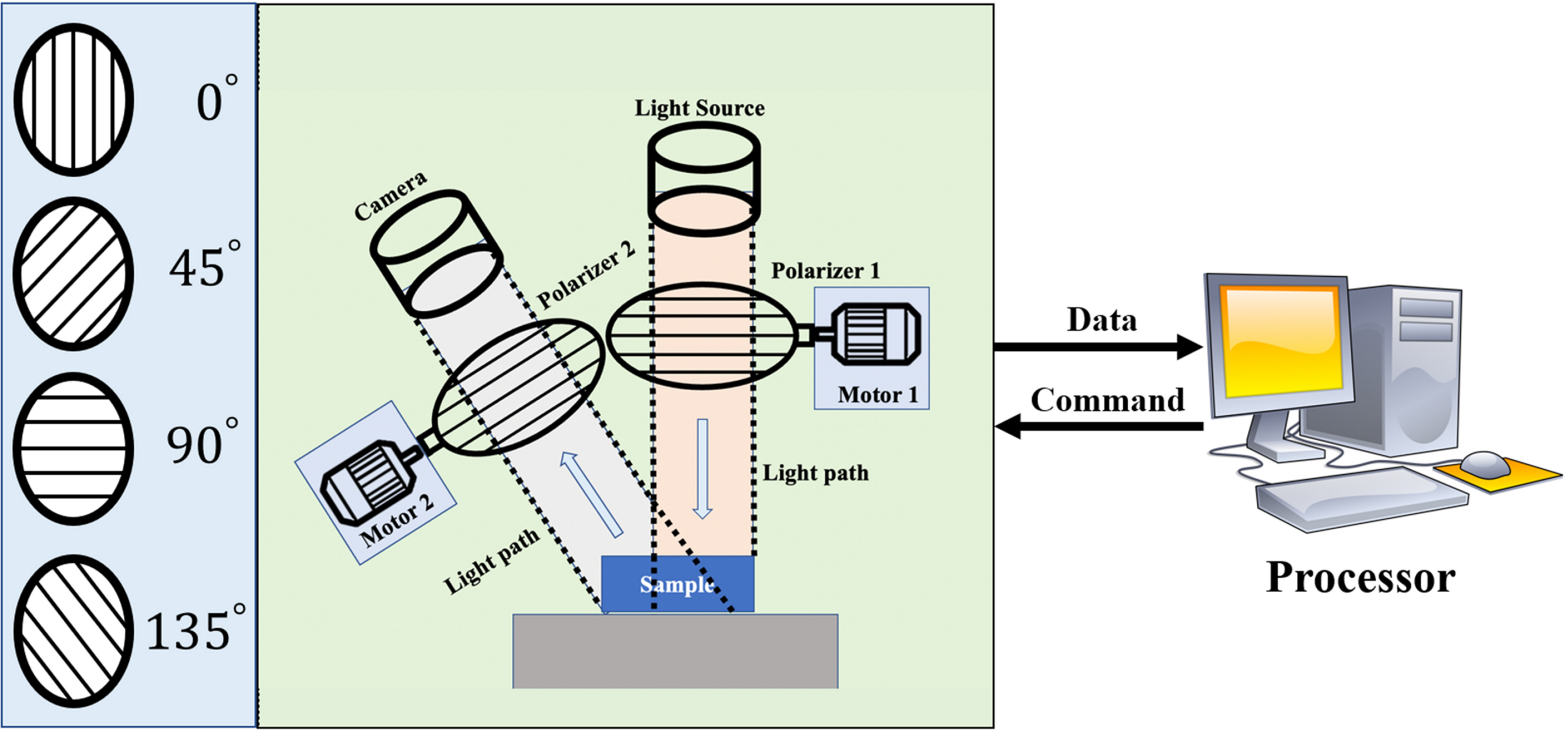
The schematic diagram of the experimental setup.

The 3×3 Mueller matrix polarization experimental system used in this study is an upgraded version of the one described earlier (39), which consisted of a light source, a PSG, an objective table, a PSA, and a CCD camera, shown in **Figure 1**. It is in backscattering mode to detect the GBM and non-GBM tissue bulk. Briefly, a collimated light source (630 nm, BT-TCL24, BTOS Telecentric Optical, China) is used to create a circular illumination area of 60 mm in diameter to illuminate the GBM and non-GBM tissue; the polarization states of the incident beam from the light source are modulated by a Polarization State Generator (PSG), and the back scattered beam with the polarization information of the GBM and non-GBM sample is analyzed with a Polarization State Analyzer (PSA). Two DC servo motors (MR-J3-40A, Mitsubishi Electric, China) rotated the polarizers covered on the driven gears to generate different PSG and PSA states, and a monochrome industry camera (MER-503-36U3M/C, Daheng Imaging, China) captures the polarization images. Through careful calibration, the maximum errors for the absolute values of Mueller matrix elements were reduced to 0.04, and the characteristic polarization information of GBM and non-GBM tissues was obtained.

### 2.2 Polarization Theory

Mueller matrix can provide samples’ abundant comprehensive microstructural information and optical polarization properties. Specifically, the Mueller matrix of the detected sample is unique, which is experimentally measured and calculated by analyzing the polarization state of the outgoing light of the sample with


$$\begin{array}{c} S_{out}=M_{sample}\cdot S_{in}\\ M_{sample}=\left[\begin{array}{ccc} m_{11} & m_{12} & m_{13}\\ m_{21} & m_{22} & m_{23}\\ m_{31} & m_{32} & m_{33} \end{array}\right] \end{array}$$

where ***S_in_*** represents the stokes vector of the incident light, ***S_out_*** represents the stokes vector of outgoing light, and ***M_sample_*** represents the Mueller matrix of the detected sample. In this paper, a 3×3 Mueller matrix, composed of the first three rows and the first three columns (40), and the backscattering configuration are used, which is particularly appropriate for *in vivo* polarimetry in clinics (34, 35).

#### 2.2.1 MMPD and MMT Methods

To solve the problem that the relationship between microstructures of the sample and the specific Mueller matrix elements is unclear, there have been several methods (36, 37, 41–46) proposed to transform the Mueller matrix into polarization parameters with clear physical meanings when it comes to the clinical applications, and several polarization parameters are proposed to characterize different features of the cancerous region. In this paper, we analyzed the PIT images of GBM and non-GBM samples and provided quantitative lines of evidence to distinguish them based on MMPD and MMT parameters: the MMPD method (37) has been widely applied to biomedical studies, and this method decomposes the Mueller matrix (***M***) into three main interactions between the polarized light and the sample: diattenuation (***D***), retardance (***δ***), and depolarization (***Δ***):


$$M=M_{\Delta }M_{R}M_{D}$$

where ***M_D_*** represents a diattenuator, ***M_R_*** represents a retarder, and ***M_Δ_*** represents a depolarizer. Since the 3×3 Mueller matrix calculation was adopted in this study, parameter ***Δ*** should be derived as follows according to Swami (37, 40):


$$\begin{array}{l} M^{'}=MM_{D}^{-1}=M_{\Delta }M_{R}\\ M_{DR}=M^{'}(M')T^{\;}\\ \Delta^{2}\cos^{2}\delta^{2}=Eig(M_{DR}) \end{array}$$

Hence, the retardance (***δ***) and depolarization (***Δ***) are obtained. In particular, the parameter ***Δ*** demonstrates the depolarization maintaining power, while (1−***Δ***) demonstrates the depolarization power (40). Furthermore, parameter ***t*** from the MMT method is calculated below, which is related to the magnitude of anisotropy for the samples (36).


$$t=\frac{\sqrt{{\left(m22-m33\right)}^{2}+{\left(m23-m32\right)}^{2}}}{2}$$

#### 2.2.2 FDH Distributions and CMCs

Using high-order statistical moments to analyze the PIT information is significant and effective when it comes to the auxiliary diagnosis for different tissues (47–49) and biological fluids (50). However, in order to extract the dominant microstructural features of the GBM and non-GBM tissues from two-dimensional images of the 3×3 Mueller matrix with rich information, we transfer the Mueller matrix images to FDHs and then calculate the CMCs of FDHs (38). The percentages of different intensity levels are given in the form of histogram of Mueller matrix elements. That is, the frequency and distribution of the same intensity level in the image. To evaluate the FDH distributions from the perspective of mathematical statistics, the CMCs, i.e., expected value (***P1***), variance (***P2***), skewness (***P3***), and kurtosis (***P4***), are calculated for a random variable X, and provide more detailed quantitative information about the sample:


$$\begin{array}{l} P1=\mu \\ P2=\sigma^{2}\\ P3=\frac{E{\left(X-\mu \right)}^{3}}{\sigma^{3}}\\ P4=\frac{E{\left(X-\mu \right)}^{4}}{\sigma^{4}} \end{array}$$

where ***P1*** is the mean value of FDH, ***P2*** is the standard deviation, ***P3*** represents the degree of asymmetry in FDH distribution, and ***P4*** represents the sharpness of the peak. Among them, ***P1*** is sensitive to the anisotropy features of the sample, ***P2*** is related to the complexity of the microstructures, ***P3*** represents the heterogeneity, while ***P4*** represents the concentration of data. Besides, the CMCs (except ***P1***) are insensitive to sample orientation directions (38). By observing the value of the CMCs, the characteristics of the FDH distribution curve are roughly obtained.

### 2.3 Human Brain Glioma Samples

**Figure 2:**
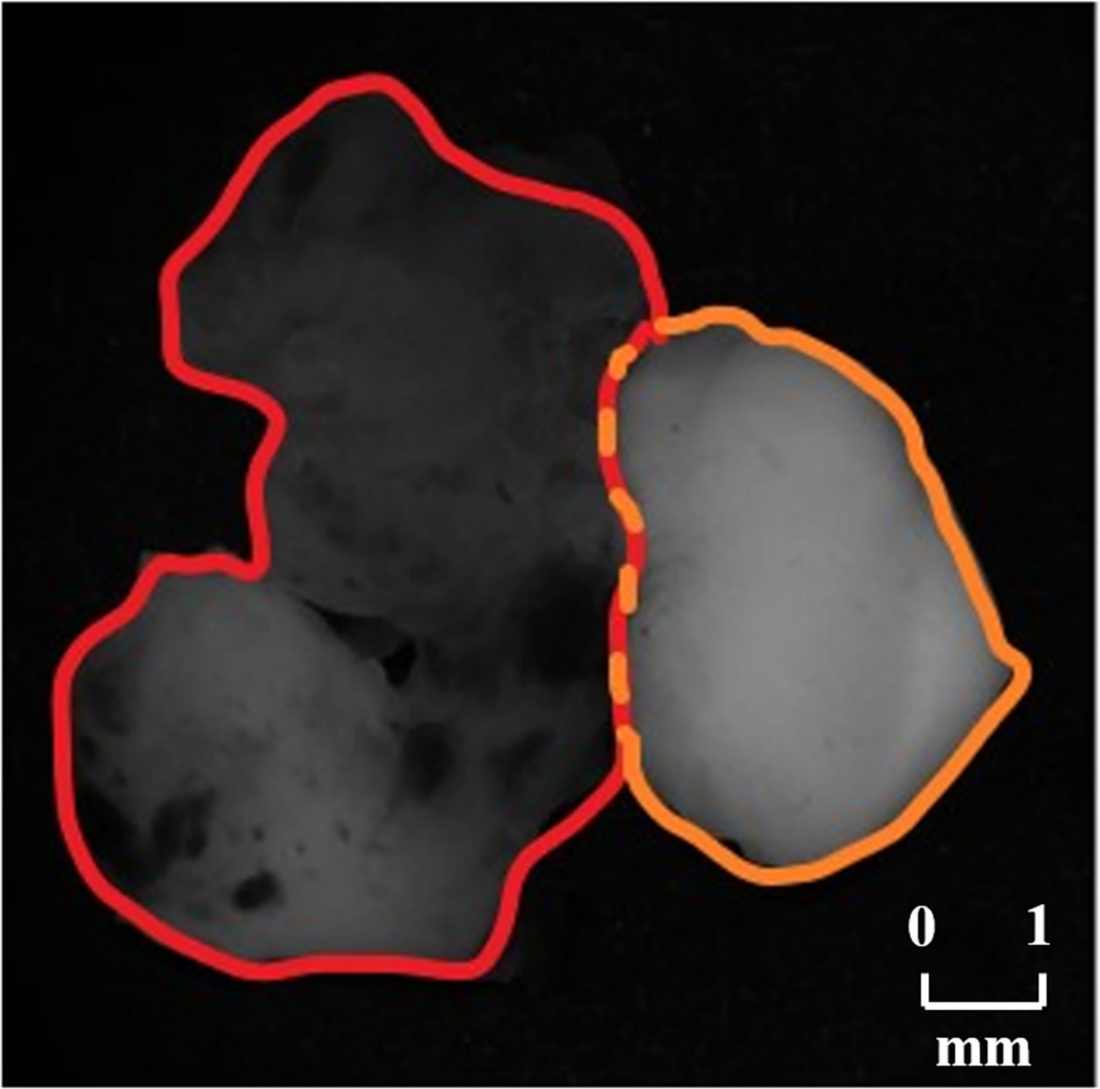
Grayscale images of the GBM sample with non-GBM brain tissue, where the GBM region is marked with red boundary delineated and the non-GBM region is marked with orange boundary delineated.

**Figure 3:**
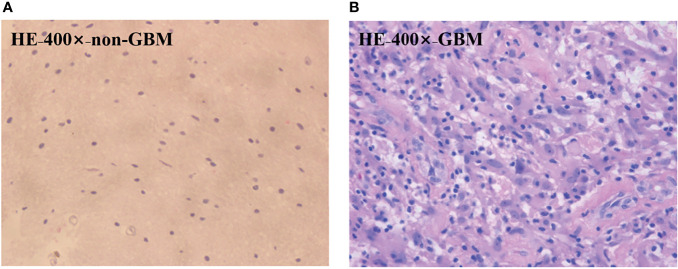
HE-stained 6-μm-thick slice of the GBM **(A)** region and the non-GBM **(B)** region from the same region of PIT images.

In order to testify the potential application of PIT on human brain glioma samples, especially when it was used to identify brain glioma from non-glioma brain tissue during surgical operation, the methods mentioned above were used on the human brain glioma samples: 20 formalin-soaked unstained GBM samples with brain white matter region (theoretically contains no glioma cells) used in this study were provided by Department of Neurosurgery of General Hospital of Tianjin Medical University, and the samples shown in **Figure 2** were grayscale image of the formalin-soaked unstained thick bulk of the GBM samples with non-GBM brain tissue cut from the patient’s cranial cavity during the surgery, with two regions marked and boundary delineated. The GBM sample is cut purely from the center of the tumor. It ensures that the polarization characterization of the GBM is reasonable, which is the first step and foundation of the further research about the identification of GBM residual during operation. However, with the imaging conditions strictly controlled, various intraoperative interference factors (such as blood) can be eliminated in *in vitro* glioma PIT experiments. For pathological comparisons, the corresponding hematoxylin-eosin (HE)-stained 6-μm-thick slices of GBM and non-GBM were also prepared and are shown in **Figure 3** to evaluate the PIT images and reveal the relationship between the pathological features and PIT images of the samples, showing that the GBM region was with a darker stained color (which means high cellular density, and denser and larger cell nuclei) than the non-GBM region. The use of the clinical glioma samples in this study was approved by the Administrative Committee on Animal Research of the Shenzhen International Graduate School, Tsinghua University. All experiments and methods were performed in accordance with the relevant guidelines and regulations.

## 3 Results

### 3.1 Characteristic Features in Backscattering Mueller Matrix Elements

Examining the characteristic behaviors of the backscattering 3×3 Mueller matrix elements of GBM samples with non-GBM brain tissue reveal rich information on the optical differences and microstructural properties, and these differences distinguish GBM from non-GBM tissues well.

**Figure 4:**
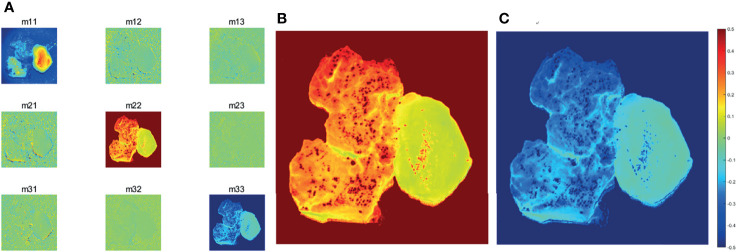
**(A)** Images of 3×3 Mueller matrix elements of detected GBM samples with non-GBM brain tissue. All the Mueller matrix elements of the sample are (except ***m_11_***) divided by ***m_11_***. **(B)*m_22_*** image of GBM samples with non-GBM brain tissue.**(C)*m_33_*** image of GBM samples with non-GBM brain tissue. The scale of the normalized elements is given by the color bar.

The structure gets disorganized in the GBM region, so its morphological and optical polarization features changed with respect to that in non-GBM brain tissue, which are directly reflected on the images and values of Mueller matrix elements. The experimental results of the backscattering 3×3 Mueller matrix of GBM tissue and non-GBM brain tissue are shown in **Figure 4** and **Table 1**. In **Figure 4**, the contrast between the sample and the background is clearly enhanced in the elements of Mueller matrix ***m_22_*** and ***m_33_*** with respect to the intensity image ***m_11_*** (at the top left corner), and the borders and detailed information in GBM and non-GBM brain regions are easily distinguished, showing the potential of the PIT for glioma detection. In **Table 1**, the average values of the Mueller matrix elements for both GBM and non-GBM tissue are listed in **Table 1**, and the characterization of PIT differences between them are clearly observed, e.g., the values of ***m_22_*** for GBM and non-GBM regions are 0.32 and 0.09, while the values of ***m_33_*** are −0.33 and −0.09. It should be mentioned that all the elements are normalized by ***m_11_***. Besides, it can be observed that these Mueller matrices were essentially diagonal, and the magnitudes of the diagonal elements are equal:


$$\left|m_{22}|=|m_{33}\right|$$

**Table 1:** Average values of 3×3 Mueller matrix elements of detected GBM samples with non-GBM brain tissues.

	*m_11_*	*m_12_*	*m_13_*	*m_21_*	*m_22_*	*m_23_*	*m_31_*	*m_32_*	*m_33_*
**GBM**	1	−0.02	−0.01	0	0.32	0	0	0	−0.33
**Non-GBM**	1	−0.02	−0.01	0	0.09	0	0	0	−0.09

Furthermore, since the magnitudes of diagonal elements are closely related to the depolarization capabilities of the samples, the non-GBM tissue has smaller diagonal ***m_22_*** and ***m_33_*** elements in **Table 1**, showing a stronger depolarization power than the GBM tissue, which are indicators to distinguish between GBM and non-GBM tissue from the perspective of Mueller matrix characterization. Combined with the corresponding pathological results shown in **Figure 3**, it is reasonable to consider that the difference in depolarization power between GBM and non-GBM tissue is related to the changes in cell density and glial fibers compared with non-GBM tissue (15).

### 3.2 Characteristic Features of PIT Parameters

**Figure 5:**
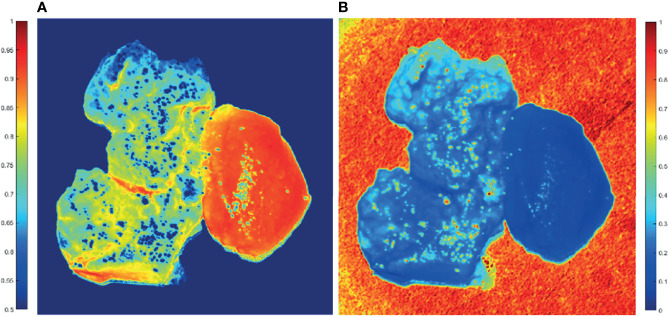
2D PIT images of PIT parameters of GBM samples with non-GBM brain tissue. The scale of the normalized elements is given by the color bar. **(A)** Parameter (1**−*Δ***). **(B)** Parameter ***t***.

Transforming the Mueller matrix into PIT parameters to separate the different effects is crucial, so MMPD and MMT parameters were calculated to characterize the PIT features of GBM and non-GBM samples. The images of parameter (1**−*Δ***) from the MMPD method and parameter ***t*** from the MMT method of GBM and non-GBM tissue are calculated and presented as follows:

From **Figure 5**, GBM and non-GBM tissue are easy to identify. Then, the average values of parameters (1**−*Δ***) and ***t*** of both GBM and non-GBM regions are calculated, which, in specific regions, remain almost the same in repeat measurements with 20 samples, and they are good indicators to distinguish a GBM region from a non-GBM region.

The average (1**−*Δ***) value of GBM region is 0.65, while the average (1**−*Δ***) value of non-GBM region is 0.91; that is, the GBM tissue (left on the image) has smaller values of (1**−*Δ***) than the non-GBM tissue. Combined with the results of the HE-stained slice of GBM and non-GBM shown in **Figure 3**, it could be that the glioma cells had a higher metabolism level, and GBM tissue contained larger density of cells and metabolism-related organelles (small scatterers, such as lysosomes and mitochondria) compared to non-GBM brain tissue caused by the uncontrolled cellular growth, resulting in a weaker depolarization power than the non-GBM tissue [smaller value of (1**−*Δ***)]. This interpretation is further supported by the results of FDH distributions and analysis of CMCs.

Meanwhile, the average ***t*** value of the GBM region is 0.33, while the average ***t*** value of the non-GBM region is 0.09; the GBM tissue has a larger value of parameter ***t*** compared with the value in the non-GBM tissue, caused by inflammatory reactions induced by carcinoma cells, which is supported by comparison with the corresponding HE-stained slices shown in **Figure 3**.

Based on these results, the differences between GBM and non-GBM tissue are identified by detecting the polarization characteristics and reflect on the PIT images and parameters. The experimental results in this section confirmed that both the PIT images and parameters (1**−*Δ***) and ***t*** are good indicators to quantitatively analyze and distinguish GBM from non-GBM tissues.

**Figure 6:**
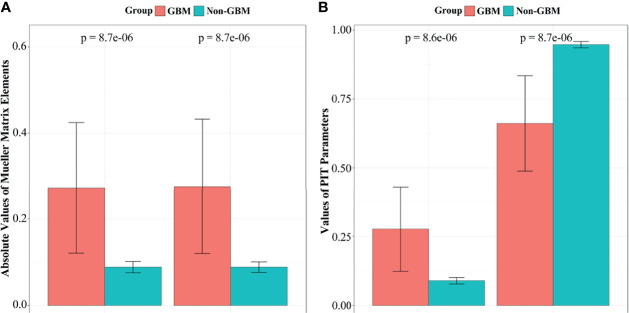
The *t*-test results and the values of corresponding ***m_22_***, ***m_33_***, parameters (1**−*Δ***) and ***t*** of GBM and non-GBM samples: **(A)*m_22_*** and ***m_33_*** elements, **(B)** parameter (1**−*Δ***) and ***t***.

Furthermore, the significance test method in the statistical *t*-test for ***m_22_***, ***m_33_***, and parameters (1**−*Δ***) and ***t*** between glioma and non-glioma tissues was used to determine the stability of the PIT characterization for GBM and non-GBM regions. *t*-tests showed a statistically significant difference (*p* < 0.05 is significant) for all the tested parameters. The *t*-test results and the values of the corresponding ***m_22_***, ***m_33_***, and parameters (1**−*Δ***) and ***t*** of 20 GBM and non-GBM samples are presented in bar charts (**Figure 6**), in which *p* is the *p*-value of GBM and non-GBM tissues of experimental data in different Mueller matrix elements and PIT parameters.

### 3.3 Features of FDH and CMCs Characterization

**Figure 7:**
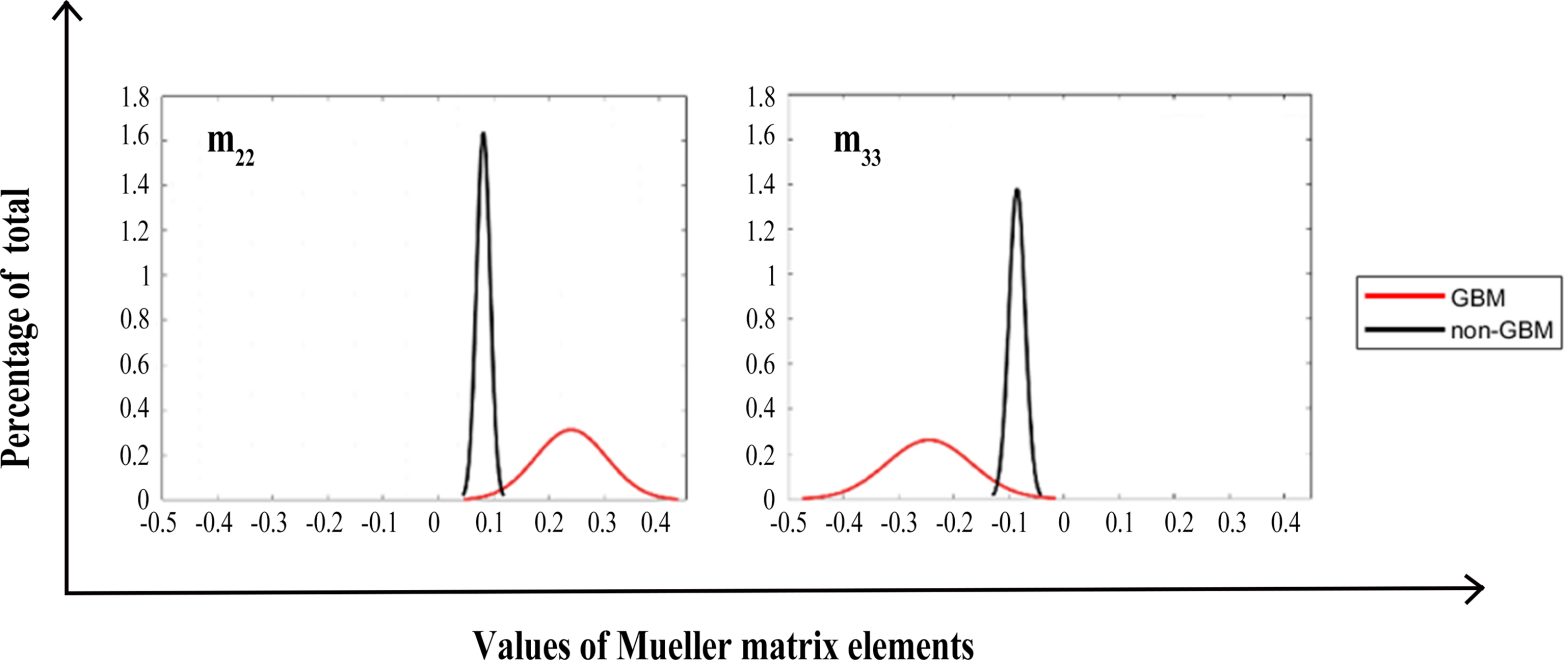
FDH distributions of diagonal Mueller matrix elements of GBM (red lines) and non-GBM tissue (black lines).

**Figure 8:**
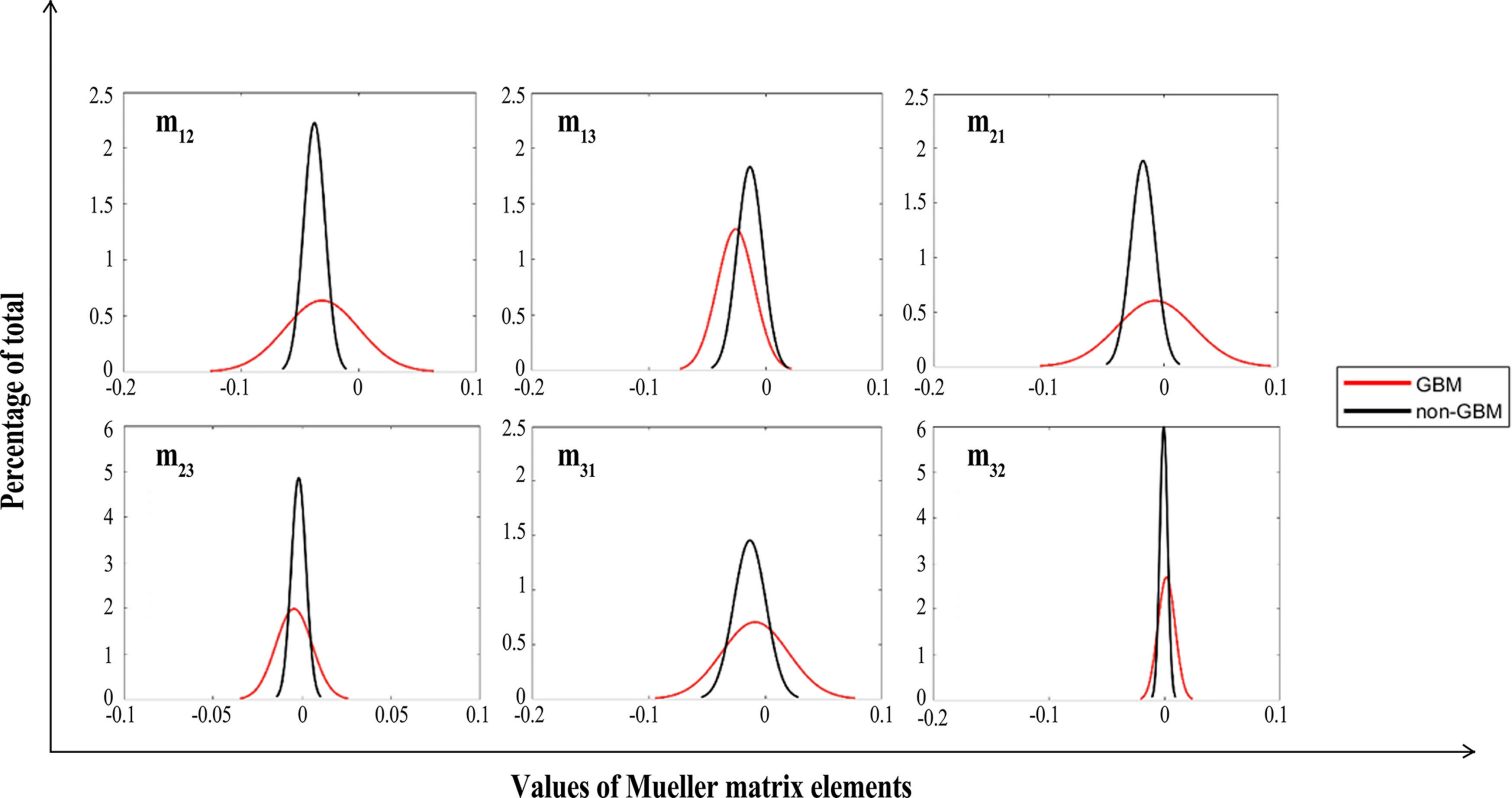
FDHs of off-diagonal Mueller matrix elements of GBM (red lines) and non-GBM tissue (black lines).

We calculated the FDHs (as shown in **Figures 7**, **8**) and CMCs (***P1***, ***P2***, ***P3***, and ***P4***, as shown in **Table 3**) of the Mueller matrix elements of the GBM and non-GBM region to extract dominant tissue microstructural features. The area under the curve of each FDH distribution is 1. The calculation of FDH and CMCs were performed on all detected samples, which had a good consistency.

As in **Figures 7**, **8**, we present the FDH results of one of those samples, and show that GBM and non-GBM tissues are easily differentiated from their FDH distributions. To be specific, for diagonal elements ***m_22_*** and ***m_33_*** of the Mueller matrix, the width of FDH distributions of non-GBM regions is smaller than those of the GBM regions, while the peak values of FDH distributions of non-GBM regions are much larger than those of the GBM regions. Furthermore, the average peak values (***h***) of FDH distributions of non-GBM regions (***h*-non-GBM**) and GBM regions (***h*-GBM**) are listed in **Table 2**, and the FDH distributions of non-GBM regions are much closer to the point of origin than the GBM regions’ FDH distributions.

**Table 2:** The average peak values (*h*) of FDH distributions of GBM and non-GBM regions.

	m22-FDH	m33-FDH
(***h*-GBM**)	0.31	0.26
(***h*-non-GBM)**	1.63	1.37

As for off-diagonal elements of the Mueller matrix, the characteristics of the FDH distributions are in great agreement with those for diagonal elements of both GBM and non-GBM regions. However, the FDH distributions of both GBM and non-GBM regions are closer to the point of origin. Theoretically, the occurrence and development of the GBM changed the scattering and absorption of the incident light, making the Mueller matrix of GBM tissue different from that of non-GBM tissue; therefore, the FDH distributions of GBM and non-GBM regions differed greatly, e.g., as for the FDH distribution of the ***m_22_*** element, the values are mostly distributed approximately 0.1 in GBM regions, while it is mostly distributed approximately 0.2–0.3 in non-GBM regions, and the peak value of FDH distributions in the non-glioma region is much larger than that in the GBM region.

The CMCs of the Mueller matrix elements of different regions are listed in **Table 3**. Firstly, the value of ***P1*** of ***m_22_*** and ***m_33_*** of GBM tissue is 3.09 and −2.79, while the value of ***P1*** of ***m_22_*** and ***m_33_*** of non-GBM tissue is 2.46 and −2.34, respectively. The absolute values of ***P1*** of diagonal elements in GBM regions are larger than those in non-GBM regions, which are in great agreement with the results presented in **Table 1**, which demonstrated the relation between the value of diagonal elements and the depolarizing power of the GBM and non-GBM samples. Secondly, the values of ***P2*** shown in **Table 3** are highly susceptible to the complexity of the microstructures in the GBM and non-GBM samples, and the values of ***P2*** of the Mueller matrix elements in non-GBM regions are significantly larger than those in GBM regions, suggesting that the non-GBM tissue including neurons and neuroglia cells is more complicated and sophisticated than the GBM tissue, which mainly contains glioma cells. Furthermore, the values of ***P3*** associated with the heterogeneity of the GBM and non-GBM samples. In the diagonal elements of the Mueller matrix, GBM regions have lower ***P3*** values so that the microstructures in this tissue are basically consistent fiber arrangement compared with non-GBM regions. As for the ***P4*** values shown in **Table 3**, it showed smaller variation in non-GBM tissue, indicating that most of the values are distributed to the mean value closely.

**Table 3:** CMCs of the Mueller matrix elements for GBM (red lines) and non-GBM regions.

	*m_12_*	*m_13_*	*m_21_*	*m_22_*	*m_23_*	*m_31_*	*m_32_*	*m_33_*
**GBM-P1**	−0.63	−1.39	−0.08	3.09	−0.02	0.02	0.28	−2.79
**Non-GBM-P1**	−1.6	−1.74	−0.21	2.46	−0.1	−0.2	0.14	−2.34
**GBM-P2**	46.99	363.33	2.32	145.07	5.15	3.34	24.21	119.13
**Non-GBM-P2**	218.19	320.17	18.42	294.46	41.7	7.33	9.04	216.06
**GBM-P3**	−0.15	−0.78	−0.16	1.14	−0.05	1.04	0.42	−1.91
**Non-GBM-P3**	−0.35	0.33	−0.43	2.06	0.22	−0.02	−0.35	−2.44
**GBM-P4**	13.55	17.8	10.54	6.47	16.78	18.46	22.87	10.56
**Non-GBM-P4**	13.47	15.32	11.31	11.88	19.32	12.92	20.69	15.15

In brief, the results confirmed that our interpretations are in accordance with the relation between the Mueller matrix elements and PIT characteristics presented in Section 3.1. The density of the cells and complexity of glial fiber in GBM and non-GBM tissue make them different in polarization properties, and reflect on the features of FDH distribution and CMC characterization, which are also indicators to distinguish between GBM from non-GBM tissue.

## 4 Discussion

In this paper, *ex vivo* PIT measurements combined with the FDH and CMC characterization of unstained formalin-soaked GBM and non-GBM tissue are performed with a 3×3 Mueller matrix polarization experimental system at a wavelength of 630 nm, and the preliminary results showed that the GBM tissue is distinguished from the non-GBM tissue. However, since this study is the first step towards realization of glioma intraoperative imaging and residual detection, it is difficult to apply in clinical applications, and the resolution of our experimental setup should be improved. There is ongoing work towards collecting fresh human glioma samples from surgery without soaking in formalin to simulate the *in vivo* and intraoperative environment, and the updated version of the PIT experimental setup is being built and intended to characterize the PIT features of the fresh glioma samples to promote the achievement of accurately identifying glioma residues intraoperatively.

## 5 Conclusions

In summary, we applied PIT to facilitate the quantitative detection of the GBM and non-GBM tissue. The 2D images of MMPD and MMT parameters (1**−*Δ***) and ***t*** of unstained GBM and non-GBM tissues were calculated and analyzed. For more quantitative comparisons, FDH distribution and CMCs were also calculated to characterize the statistical differences in the 2D images of Mueller matrix elements of GBM and non-GBM tissue. The experimental results indicated that, although more detailed information was needed, PIT images and retrieved parameters may have potential to be indices that can distinguish glioma from non-glioma tissues. This paper demonstrated the potential of using the PIT method for glioma detection during surgery and showed a good diagnosis application prospect.

## Data Availability Statement

The original contributions presented in the study are included in the article/supplementary material. Further inquiries can be directed to the corresponding author.

## Ethics Statement

The studies involving human participants were reviewed and approved by the Administrative Committee on Animal Research of the Shenzhen International Graduate School, Tsinghua University. The patients/participants provided their written informed consent to participate in this study.

## Author Contributions

Y-RL carried out the experiment, performed the data analysis, and was responsible for the writing of the manuscript. JW and H-HH supervised the work. All authors contributed to revising the manuscript and approved the final version.

## Funding

This work was supported by the Overseas Research Cooperation project of Tsinghua Shenzhen International Graduate School (HW2018005), the National Key R&D Program of China (2019YFC0119500), and the Knowledge Innovation Program of Basic Research Projects of Shenzhen Grant (JCYJ20160428182053361 and JCY20200109142805928)

## Conflict of Interest

The authors declare that the research was conducted in the absence of any commercial or financial relationships that could be construed as a potential conflict of interest.

## Publisher’s Note

All claims expressed in this article are solely those of the authors and do not necessarily represent those of their affiliated organizations, or those of the publisher, the editors and the reviewers. Any product that may be evaluated in this article, or claim that may be made by its manufacturer, is not guaranteed or endorsed by the publisher.
